# From Model to Practice: A Qualitative Study on Factors Influencing the Implementation of the Active Recovery Triad (ART) Model in Long-Term Mental Health Care

**DOI:** 10.3390/jcm13123488

**Published:** 2024-06-14

**Authors:** Lieke Zomer, Lisette van der Meer, Jaap van Weeghel, Guy Widdershoven, Yolande Voskes

**Affiliations:** 1 Department of Ethics, Law and Humanities, Amsterdam University Medical Centers, 1081 HV Amsterdam, The Netherlands; 2 Altrecht GGZ, 3705 WC Zeist, The Netherlands; 3 Department of Clinical & Developmental Neuropsychology, University of Groningen, 9712 TS Groningen, The Netherlands; 4 Department of Rehabilitation, Lentis Psychiatric Institute, 9470 AC Zuidlaren, The Netherlands; 5 Tranzo Scientific Center for Care and Wellbeing, Tilburg University, 5037 DB Tilburg, The Netherlands; 6 Impact Care Group, GGz Breburg, 5017 JD Tilburg, The Netherlands

**Keywords:** recovery, long-term mental health care, implementation, qualitative methodology, rehabilitation

## Abstract

**Background**: The Active Recovery Triad (ART) model provides a framework for recovery-oriented care in the long-term mental health setting. The aim of this study is to gain insight into factors influencing the implementation process of the ART model. **Methods**: Focus groups were conducted with fourteen multidisciplinary teams that were in the process of implementing the ART model. Data were thematically analyzed. **Results**: Three phases of implementation were identified. In the first phase, getting started, support from both the top of the organization and the care workers, sufficient information to care workers, service users, and significant others, and creating momentum were considered crucial factors. In the second phase, during implementation, a stable team with a good team spirit, leadership and ambassadors, prioritizing goals, sufficient tools and training, and overcoming structural limitations in large organizations were seen as important factors. In the third phase, striving for sustainability, dealing with setbacks, maintaining attention to the ART model, and exchange with other teams and organizations were mentioned as core factors. **Conclusions**: The findings may support teams in making the shift from traditional care approaches towards recovery-oriented care in long-term mental health care.

## 1. Introduction

In recent decades, the concept of recovery has become an important guiding principle in mental health care [1,2,3,4,5]. As of 2008, the Convention on the Rights of Persons with Disabilities (CRPD) underlined the necessity and obligation of changing attitudes towards mental health care and improving the quality of care under international human rights law, leading to the World Health Organization (WHO) comprehensive mental health action plan and the WHO QualityRights initiative [6,7,8]. In the past decade, there have been several attempts to implement recovery-oriented care that either explicitly or implicitly incorporates the WHO vision regarding mental health care [8,9,10,11]. However, these attempts were mainly focused on outpatient and acute care, and only moderate improvements were established in long-term mental health care [12,13,14,15]. Long-term mental health care seems to be a complex setting to make a shift from traditional approaches towards recovery-oriented care [9,16,17]. Traditional approaches are based on the biomedical model, focusing on stabilization, controlling symptoms and dysfunctional behavior [15,17]. Recently, the Active Recovery Triad (ART) model was developed in a collaborative process including stakeholders from various mental health care organizations in the Netherlands [18,19]. The aim is to provide a guiding framework for recovery-oriented care in the long-term mental health care setting. Teams in this setting provide 24 h care and support to people with serious mental illnesses, situated at clinical wards (open or closed), residential facilities at institutional grounds, or sheltered housing facilities in the community. Service users in the long-term setting are people with complex needs due to a serious mental illness influencing their daily and social functioning. The majority are diagnosed with schizophrenia or a psychotic disorder [20,21]. In addition, other common diagnoses are bipolar disorder, personality disorder, developmental disorder, addiction, severe depression, and anxiety. Often people have coexisting problems, for example, a combination of these disorders [22], cognitive problems, somatic problems [23,24], and functional disabilities. This group of people is in need of care and support in multiple life domains, and outpatient care is considered not to be sufficient.

The content of the ART model is extensively described elsewhere [18,19]. Table 1 displays the core principles of the model. The core principles of the ART model are Active, Recovery, and Triad. First, the principle ‘Active’ involves an active attitude of service users, significant others, and care workers in order to foster the recovery process. An active attitude is fostered by a recommended length of stay [25]. During the development process of the ART model, consensus between the participating stakeholders was reached upon a timeframe of three years. Although recovery is a personal process and varies between individuals, three years was considered long enough to work actively on recovery goals, but short enough to prevent hospitalization [18]. This is not a definite limit regarding staying in the service, but a thorough evaluation should take place after this time. That is, if after these three years, the service user has not (yet) been able to reach his/her recovery goals (often including living status), a critical evaluation and reconsideration of goals, treatment, and necessary support are needed to continue care and support for another three years. The principle ‘Active’ also implies carefully taking into account personal wishes regarding treatment, support, and living. Second, the principle ‘Recovery’ entails a focus on different dimensions of recovery. These include the recovery of health, the recovery of identity, the recovery of daily functioning, and the recovery of community functioning [26]. Third, the principle ‘Triad’ entails the collaboration between service users, their significant others, and the care workers involved. Peer workers and family peer workers can represent these perspectives within the team. The triad should also be visible on the organizational level, which means that the perspectives of service users, significant others, and care workers are represented in management and policy processes. The core principles of the ART model are operationalized in a model fidelity scale, the ART monitor [27]. Teams can use this instrument to examine the degree of compliance to the ART model and as a framework to improve their quality of care.

The implementation of new approaches such as the ART model in mental health care is a challenge [10,28,29,30]. Often, there is a gap between evidence-based or effective care and routine mental health practice [29,31,32,33]. The literature focusing on implementation processes in mental health services describes several hampering factors on the organizational level (such as a lack of resources, physical structures, and staff turnover) as well as on the individual level (such as limited knowledge about the intervention, a lack of collaboration, and resistant attitude towards the intervention) [9,10,34,35]. However, the literature on implementing new approaches in long-term mental health care mainly addresses specific recovery interventions, instead of a broad and integrated framework such as the ART model.

The implementation of the ART model in Dutch long-term mental health care is also challenging. In a previous study, we demonstrated that the degree of compliance to the ART model varies between teams; some teams are further along and more successful in this process than others [27]. To understand these differences and disentangle factors influencing the implementation process, it is important to study experiences within different teams and different organizations. This paper aims to get insight into experiences within teams during the first three years of the process of implementing the ART model. The research question for the current study is as follows: What factors influence the implementation process of the ART model in long-term mental health care?

## 2. Materials and Methods

### 2.1. Study Design

This study was part of a larger research project on the implementation of the ART model and the evaluation of the ART monitor [27]. The teams that participated in the evaluation study were also approached for the current study. In order to gain insight into the views and experiences of care workers regarding the process of implementing the ART model, qualitative research is recommended [36,37]. Therefore, a qualitative design was used in this study and data were collected through focus groups with care workers. The focus groups were semi-structured, based on a topic list addressing issues relevant to the implementation process. During the focus groups, several care workers from the same team exchanged views and experiences and jointly reflected on the implementation process of the ART model. The findings are reported in line with the Standards for Reporting Qualitative Research (SRQR) [38].

### 2.2. Participants

A purposive sampling strategy was used to recruit participants [39]. Fifteen multidisciplinary teams operating in Dutch long-term mental health care, that were in the process of implementing the ART model into practice and already participated in the evaluation study of the ART monitor [27], were also invited to participate in this study. Care workers from at least three different disciplines were required to be present during the focus groups to ensure the inclusion of different perspectives in the team. Because the focus groups took place parallel to a larger study, the timing varied with respect to the implementation process of the participating teams. Six participating teams were at the start of their implementation process. In this way, we were able to retrieve information on the approach that was chosen for the implementation process, and the focus was on how they planned to start, what preparations they had already made, what they had already faced by this stage, and their future expectations. For the other eight participating teams, the duration of the implementation process of ART varied from six months to three years.

### 2.3. Data Collection

Focus groups were held with teams in the process of implementing the ART model. From every team, multiple team members participated, in order to discuss views and experiences and reflect on successes and challenges. Prior to data collection, a topic list was developed (Supplementary Materials S1). Topics included the timing and strategy of the start of the implementation process; steps that have been taken; the successes and challenges in the process; future expectations; and advice to other teams concerning the implementation process of the ART model. The focus groups took place between June 2018 and April 2019. The first author of this study (LZ) conducted the focus groups. The duration of each of the meetings was approximately one hour, and they took place at the location of the participating teams. With the permission of the participants, all focus groups were audio-recorded for analysis.

### 2.4. Data Analysis

Data collection and analysis were performed simultaneously. First, a preliminary analysis of each of the focus groups took place as the basis of a report for the participating teams. That is, a summary of the main topics of the focus group was written and sent to the team. This also enabled us to establish that data saturation was reached at the end of the process of data collection. Second, further analysis of the data was performed by means of a thematic analysis [37]. After transcribing the focus group recordings and familiarizing ourselves with the data, transcripts were coded using MAXQDA version 2020 [40]. Two researchers (LZ and YV) discussed the codes to check whether similar codes were identified (triangulation). When different codes were labeled, they were discussed until consensus was reached. Subsequently, similar codes were clustered into provisional themes which reflected factors influencing the implementation by two researchers (LZ and YV). Based on the provisional themes that were identified, a categorization was made in three phases of the implementation process, namely, (1) getting started, (2) during implementation, and (3) striving for sustainability. The provisional themes were discussed among all authors and refined in view of coherence and distinctiveness until consensus was reached. Titles were chosen for each theme and discussed among the authors (see Supplementary Materials S2 for the coding tree). Also, the process of implementation in one team with high ART monitor scores (as reported in [27]) was described in detail as an ‘illustrative case’, describing events and experiences for each of the three phases.

### 2.5. Researcher Characteristics and Reflexivity

The research team includes experienced qualitative (and quantitative) researchers. Central topics in the research of all authors are recovery, rehabilitation, participation, and the reduction of coercion in psychiatry. Three authors (LvdM, JvW, and YV) were involved in the development of the ART model. After the model was developed, the first author (LZ) performed research on the implementation and impact of the ART model, as part of her PhD. She received training on performing qualitative research during her Master’s program (Management, Policy Analysis and Entrepreneurship in the Health and Life Sciences at the VU in Amsterdam), and as part of her PhD, she followed an advanced course on qualitative research methods and on mixed-methods. LZ conducted the focus groups, under the supervision of YV and LvdM. Data were coded by two researchers (LZ and YV) and analyzed in two steps, first by two researchers (LZ and YV), and later by the whole team.

### 2.6. Ethical Considerations

This study was approved by the Medical Ethical Committee and the Scientific Quality Committee of Amsterdam UMC. All participating team members received an information letter about this research prior to the start of the study. At the start of the focus groups, the first author (LZ) explained the aim of the study to the participants and asked for verbal consent. After the focus groups were conducted, a summary of the main points was written, as part of a report for the team, and sent to the team for a member check. Thereby, members of the team had the opportunity to suggest revisions to the summary of the focus group. However, none of them indicated any revisions.

## 3. Results

First, participant characteristics will be provided. Next, the case of one team which was advanced in the process of implementing the ART model will be presented, focusing on the three phases of implementation. Thereafter, we will describe the factors that were identified across sites, also ordered according to the three phases of implementation.

### 3.1. Participant Characteristics

Fourteen teams, all from different organizations, participated in this study. For one team that was approached, it was not possible to organize the focus group because of the lack of time and lack of availability of three different disciplines at the time of the focus group. Seven teams provided care at long-stay open wards, two teams at partially open/partially closed settings, one team at a long-stay closed ward, and four teams at housing facilities located on institutional grounds. In total, 83 participants from various disciplines were included in this study; see Table 2.

### 3.2. Illustrative Case of Team X

Table 3 presents the course of implementation of a team in the process of implementing the ART model. We refer to the team as team X. We will describe their experiences in detail for three phases of the implementation process; getting started, during implementation, and striving for sustainability.

### 3.3. Factors for Phase 1: Getting Started

For the first phase of the implementation process, getting started, the three following factors were identified: (1) support from the management and the work floor, (2) providing information to care workers, service users, and significant others, and (3) momentum.

#### 3.3.1. Support from the Management and the Work Floor

Support from management and the work floor appeared to be important during the start of the implementation process. The initiation of implementing the ART model differed per team and organization. In some organizations, the initiative to introduce the ART model came from the management of the organization. For other participating teams, the initiative was taken by care workers from the work floor who found out about the development of ART. Ideally, both approaches are combined:

“*I think it was about three years ago, the development of the ART model had just started. I saw a message on Twitter. I looked it up, I saw some key words and I thought wait a minute, that’s something we could really use at our ward. Then I went to my team leader to ask her what she thought of it, and she was also very enthusiastic. We went to the first conference with three or four people. We came back very enthusiastic. At that time the ART handbook still had to be written. I was asked to participate in this*.”

(-nurse, FG 6)

Participants indicated that it is a challenge when either the management of the organization or the professionals on the work floor do not support the introduction of the model. A nurse practitioner said:

“*We are the only team in our organization that works with ART. Sometimes I have the feeling the management does not fully support the vision. Sometimes I have the feeling we are doing it alone*.”

(-nurse practitioner, FG 3)

In the case of team X, the director of the organization took the initiative, but he involved several care workers from the work floor into the development process of the ART model. Participation in the development process by various stakeholders from the organization was indicated during several focus groups as a success factor to create broad support. A nurse explained:

“*Two of our team members took part in some meetings during the development of the ART model. Since that time, we tried out some parts of the model. I think this helped us in the start and moving forward*.”

(-nurse, FG 8)

#### 3.3.2. Information to Care Workers, Service Users, and Significant Others

Providing the opportunity for care workers, service users, and significant others to get acquainted with the ART model was regarded as important at the start of the implementation process. Often, care workers read the ART handbook and, in some organizations, the authors of the ART handbook were invited to give a presentation about the ART model. Within the organization of team X and some other participating organizations, a first ‘kick-off’ meeting was set up to provide information about the ART model. However, for some people the content of the ART model was not clear:

“*The concepts related to recovery-oriented care and the ART model are quite new for some colleagues and not clear for everyone in our team. Some people are quite skeptical about the ART model. It would be nice if we could receive more explanation, besides only the ART handbook*.”

(-social worker, FG 9)

Furthermore, some care workers indicated that the ART model is not something new. A nurse stated:

“*I work in this setting for 17 years. I have the feeling we have already worked in line with the ART model for a long time. However, now concepts get another name*.”

(-nurse, FG 14)

Moreover, several participants emphasized the need to involve service users and significant others from the start. Therefore, they were sometimes invited to kick-off meetings, or separate information meetings for service users and significant others were organized:

“*We organized a meeting for service users and family members to explain what the ART model is and provide information on what this implies for them. We think it is very important to include service users and* family*, so they can think along with us. And I think repetition is very important, so we will organize a second meeting soon*.”

(-care coordinator, FG 7)

Some participants mentioned that the guided maximum length of stay of three years, as stated in the ART model, created insecurity and resistance among service users, their significant others, and care workers. A psychiatrist said:

“*The limited timeframe of the ART model has an impact on everyone, especially on service users and family. Family indicated to us: ‘he has been here for 10 years, where is my relative going? Wasn’t he allowed to stay here?’ That has also been said to many people, you go to* [name of ward] *and you can stay there. Also, I do think there might be a small group of people who remain dependent on these long-stay facilities, for whom this timeframe of three years is not helpful*.”

(-psychiatrist, FG 1)

To overcome such resistance among service users and family members, several participants stressed the importance of investing time and effort to inform and explain the ART model to service users and significant others, specifically in the individual contact with service users and significant others.

#### 3.3.3. Momentum

To get the implementation process going, momentum is needed. For some of the participating teams, including team X, the kick-off meeting provided momentum. Other teams created momentum by organizing a team-building day or receiving an ART audit as a baseline measurement to start the implementation process. This fostered energy and enthusiasm among care workers to start the implementation process. A nurse practitioner, working in a team where momentum was lacking, indicated that the implementation process remained ambiguous and noncommittal in the beginning:

“*We did not define a starting point. We did not say: ‘December 1st we will start…’ We only included the goal of implementing the ART model in our year plan. This made the implementation process very noncommittal. We read the ART handbook and thought about it, but it was never a structured process*.”

(-nurse practitioner, FG 3)

### 3.4. Factors for Phase 2: During Implementation

We identified five factors as important during the implementation process, namely, (1) a stable team with a good spirit, (2) leadership and ambassadors, (3) prioritizing goals, (4) sufficient tools and training, and (5) overcoming structural limitations in large organizations.

#### 3.4.1. A Stable Team with a Good Spirit

A stable team including care workers that support the vision of the ART model was regarded by participants as necessary for the implementation. However, for most participating teams, including team X, this was challenging. All participating teams struggled with a shortage of staff, a high turnover, and difficulties in finding new employees.

“*Recently we have a really high turnover. You need a fairly stable team to jointly develop a new way of working. That is not possible when care workers are leaving constantly, as you lose the knowledge and experience when people are leaving*.”

(-social worker, FG 14)

During some focus groups, participants mentioned that the setting of long-term mental health care is not perceived as an attractive working environment to (mostly younger) employees, because, for a long time, the type of work in this setting was not focused on recovery, but rather (clinical) stability. However, participants indicated to be hopeful that the ART model would provide a new impulse. A psychologist said:

“*When you tell people that you work in long-term mental health care, people don’t think it’s an attractive setting to work in. The image is that people live there, and somatic care is the only thing you can provide. I think we should express more how interesting our setting actually is. And how much you have to think out-of-the-box. How many great things you can achieve. Yes, a large change certainly won’t be achieved in three weeks. But what you can achieve in the end and how much added quality of life you can give to someone, that is actually what I love the most in my work*.”

(-psychologist, FG 1)

A positive atmosphere and good cooperation in the team were indicated in several focus groups to be helpful in implementing the ART model. Investment in team spirit by means of team (-building) activities, meetings, and time for reflection were indicated to contribute to a positive atmosphere and good cooperation. It is important to have everyone on board, supporting the vision of the ART model. A manager explained her strategy:

“*Our team members have explicitly chosen to work with ART in the team. At the start of the implementation process, all professionals in the organization have been asked to indicate where they would like to work and, based on this, they were allocated to the different teams. In our team, we collectively chose to implement the ART model*.”

(-manager, FG 13)

#### 3.4.2. Leadership and Ambassadors

To stimulate the implementation process, leadership based on mutual trust and working with ART ambassadors were regarded as helpful. It is important that team members feel encouraged and have time to work on innovations, acknowledged by and together with the management and treatment staff of the team. In addition, several participants indicated that ambassadors or project leaders for ART can play a prominent role in the implementation process:

“*We are the ambassadors, my team includes older colleagues who find it difficult to keep the overview. They have all read the handbook and support the vision, but they find it difficult to put it into action.* […] *We get support from* [NAMES MANAGERS], *everything we ask is approved. They also see that things are going well. We only have to keep an eye on the process and encourage everyone*.”

(-care coordinator, FG 7)

In the case of team X, the ambassadors were nurses or social workers in the team that advocated and promoted the ART model, took the lead in new developments, and had contact with ambassadors of other teams. Ambassadors often volunteered, were selected by the management, or, for example, became ambassador because they also obtained the role of auditor in the national ART research. However, a risk is that only these ambassadors feel responsible for the implementation and the rest of the team is left out in this process:

“*I struggle in my role* [as ART ambassador in the team]. *Sometimes I have the feeling that I am responsible for implementing ART on my own. I try to involve my colleagues by sharing my experiences with the audits I performed and share the lessons I have learned. But it is difficult to get the whole team on board, because everyone is very busy*.”

(-nurse, FG 3)

#### 3.4.3. Prioritizing Goals

Prioritizing goals was indicated as important for the implementation process. As one peer worker reflected:

“*It is impossible to implement all elements of the ART model at once*.”

(-peer worker, FG 7)

Many participating teams selected a limited number of items from the ART monitor to start with. Usually, these items were selected based on low scores on the ART monitor or because the content of these items appealed to the team members. A team leader said:

“*It is important to set small goals, actually similar to the small steps service users take in their recovery process. You have to do this together, to decide upon these goals together with the team and have the support of all colleagues*.”

(-team leader, FG 2)

Like team X, some other teams also worked with a structured process to set a deadline to achieve a goal, evaluate this achievement, and set new goals. One participating team developed a tool to make the implementation process visible for everyone:

“*We have put a board in our office with five boxes corresponding to the scores of the ART monitor. On this board, we write down all the items from the ART monitor, including the score we have at that moment. Everyone in the office can see this and colleagues can pick up an item they would like to start with. During our team meetings we evaluate this, and see whether we can achieve a higher score for the items on the board*.”

(-social worker, FG 13)

#### 3.4.4. Sufficient Tools and Training

Making use of tools and training were regarded as important during the implementation process. First, the ART monitor and ART handbook were perceived as helpful tools:

“*The great thing is that you have a handbook that gives a direction to recovery-oriented care, which we have been working on for a long time. Then you can review the ART monitor and you are able to see: oh yes, we already have that, oh yes, we are going to do that, we don’t have that*.”

(-peer worker, FG 7)

Care workers in several focus groups mentioned having received training on subjects related to the ART model. These trainings contributed to theoretical and practical knowledge, for example, on how to support the recovery process of service users or how to support and collaborate with significant others. However, there were also some challenges related to training. A manager said:

“*We wanted to have a mix in how we offer the trainings. Because when care workers follow a training or education outside the work environment, it is sometimes hard to adopt the things that they have learned into practice*.”

(-manager, FG 5)

Therefore, this team received coaching on the job regarding the ART model and recovery-oriented care. Another team received support from a worker in the organization specialized in the development and implementation of new methods. Besides the training on specific subjects, the need to develop a training program on the ART model as a whole was mentioned as important in various focus groups.

#### 3.4.5. Overcoming Structural Limitations in Large Organizations

Overcoming the limitations of existing structures in large organizations appeared to be an important theme in the implementation process of the ART model. Several participating teams, including team X, are part of large organizations. Often, the structures of these large organizations hinder the pace of implementation. One example was provided by a nurse:

“*The clinical record we work with is very outdated. The primary focus is on medical aspects, and we have no space to report information on other aspects of recovery. Our clinical record does not match with the principles of the ART model*.”

(-nurse, FG 14)

Another example is the outdated housing facilities or wards the teams operate in, which are often small, have shared accommodations, and are sometimes in a poor state. It requires the creativity and flexibility of team members and service users to innovate and improve quality of care within such an environment. A manager said:

“*We have to look at the facilities of our ward and the way we work. We have to be creative and work in a completely different way. That may mean that things don’t necessarily go the way we always did. In psychiatry we are good at developing structures that are not always helpful*.”

(-manager, FG 5)

### 3.5. Factors for Phase 3: Striving for Sustainability

The third phase of the implementation process regards striving for sustainability. We identified three factors, namely, (1) dealing with setbacks, (2) maintaining attention to the ART model and (3) Exchanging with Other Teams.

#### 3.5.1. Dealing with Setbacks

Dealing with setbacks was regarded as important, especially later in the implementation process. Setbacks teams faced were, for instance, related to the shortage of staff:

“*Because we have so many open vacancies, it is difficult to keep the implementation process alive. I must say, the progress in the implementation process is not as fast as we expected*.”

(-manager, FG 5)

Other limitations were a shortage in accommodation when service users were ready to move to a place with less support or financial issues resulting in hardly any room for attracting new personnel or performing activities contributing to the implementation process. Team X clearly struggled with a shortage of personnel and financial issues, which hindered the implementation process. An important strategy by which teams maintain positive energy is to keep on sharing and celebrating (small) successes that have been achieved. In addition, the importance of stimulating service users to motivate each other was stressed by a peer worker:

“*Service users started to share experiences in a group and learn from each other. An example from last week was the discussion around the subject of friendship.* […] *A service user shared an experience about the buddy project. There were many positive reactions from other service users. This also gives us energy as a team to continue working on this*.”

(-peer worker, FG 2)

#### 3.5.2. Maintaining Attention to the ART Model

The difficulty of maintaining attention to the ART model was stressed in several focus groups. Different strategies were indicated. First, an external audit was mentioned by several teams:

“*We see this external audit as an incentive not to lose our attention. What do experienced auditors, who have experience with implementing ART themselves, say about where we stand in the process? What are their recommendations? What should we focus on? The audit report and the recommendations we have received really provide guidance in that*.”

(-nurse, FG 8)

Second, in some organizations, internal audits were organized for all the teams working with the ART model. Third, participants of several teams indicated to benefit from planned meetings, either with their own team or together with other teams from the organization, to evaluate the implementation process. The management of team X organized these meetings every six months with all teams that implemented the ART model together, to share experiences and successes. Last, attending the annual ART conferences and platform meetings was also indicated to be contributive to obtaining new energy and inspiration for the implementation process. A social worker said:

“*We always go to the ART conferences and other national meetings. Of course, every time with different colleagues, as we want to give everyone the opportunity to visit these meetings*.”

(-social worker, FG 13)

#### 3.5.3. Exchanging with Other Teams

The exchange of knowledge and experiences with other teams within the organization, as well as with teams from other organizations, was perceived as important for the sustainability of the implementation process. A team leader provided an example of this:

“*There is currently a pilot project to stimulate exchange between teams, in which colleagues temporarily switch workplaces. For example, a worker of a closed ward works for a month in a completely different setting, such as a sheltered living location, and vice versa*.”

(-team leader, FG 2)

The management of team X initiated several ways to exchange knowledge and experiences between the different teams in the organization. In several focus groups, the existence of a national learning network was mentioned as inspiring:

“*The fact that you all work on the same thing and you all share the same vision, you really receive a boost from this. During the conference you notice that teams from other organizations in the country also want to go for it. That creates a connection. You hear stories from other teams, no matter how different their ward or location is. You notice the similarities. This also inspires us, and we can use these insights to take a step further in our own process*.”

(-nurse practitioner, FG 4)

## 4. Discussion

The aim of this study was to create insight into factors influencing the implementation of the ART model. We identified three phases of implementation and a total of eleven factors. The case of team X served as an illustration of how these factors influence the course of implementation within a team operating in Dutch long-term mental health care.

The three phases of implementation which we distinguished reflect phases which are described in the literature. Some authors mention four phases, that is, exploration, installation, initial implementation, and full implementation [41]. The first two of these are combined in our first phase, getting stated. The Quality Implementation Framework also contains four phases: initial considerations regarding the host setting, creating a structure for implementation, ongoing structure once implementation begins, and improving future applications [42]. The first of these phases is not applicable to our study, as the process of implementation which we investigated was initiated by the organizations themselves. The other phases mirror our categorization.

The factors we identified for the various phases each contain several facilitators and barriers. Facilitators were to a large extent in line with the literature and comparable with other implementation processes in (mental) health care. Examples are support from both the management of the organization and the work floor [43,44], momentum [45], a stable team [46], time to invest in the team spirit and work together on innovation [47], leadership [44,48,49,50], ambassadors [34,51], extra tools and trainings [52,53], and the exchange of knowledge and experiences with other teams [52]. This study only covered the first three years of implementation of the ART model, so other facilitators described in the literature may be relevant for the sustainability of the implementation over a longer period of time, for example, the collaboration with other partners, such as stakeholders in the community [54], the ongoing supervision of staff [34,55], and adequate funding [55].

Most of the barriers we identified were related to the specific context of long-term mental health care. Insecurity and resistance regarding the ART model among service users and significant others appeared to be specifically related to the context of long-term mental health care. That is, the introduction of the ART model has a large impact on service users; for a large group, this implies a shift from receiving inpatient care for decades to thinking about their own wishes and goals for the future and working on these goals within a timeframe of three years. The ART model also required changes in the role of family and significant others, from being left out of the care process to protect them or having no contact at all, to being a partner in an active collaboration in which their knowledge and experiences are of crucial importance. Taking the time to involve service users and significant others in the process helped teams to talk about and overcome this barrier. Other barriers, for instance, the shortage of staff, have also been described for other contexts, such as acute mental health care [56] and community mental health care [48,49,57]. However, the image of the long-term mental health care setting appears to be a significant problem in attracting new employees. The ART model might lead to a shift in how this setting is regarded, from a last resort for most service users to a recovery-oriented perspective including hope and a focus on possibilities. The results of this study also point out that some care workers are skeptical regarding the ART model, and not everyone supports the vision of the ART model. The ART model requires a different daily working routine and other competencies of professionals, from taking care of and taking over from service users to a coaching and supporting role. This is in line with other studies on recovery-oriented care and the change in the competencies and skills of care workers [58,59].

Unique for the context of the ART model in Dutch long-term mental health care was the collaborative development process on a national level, that contributed to a broad support among care workers, service users, and significant others. Also, various activities that were organized, such as audits, ART conferences, and platform meetings, offered the opportunity to share knowledge and experiences. As a result, a learning community [60,61] or community of practice [62] was created. Learning communities in general appear to enhance the sustainability of evidence-based practices, as in the example of the IPS (Individual Placement and Support) learning community [61]. Following the developments of the ART model over time may show long-term effects, as, in this study, we only covered the first three years of implementation and working with this community of practice.

### Strengths and Limitations

This study provided insight into experiences of team members regarding factors influencing the implementation of the ART model in long-term mental health care. It also contributed to the implementation process of the ART model, by creating a moment for team members to jointly reflect on the process they had been through, challenges they had faced during this process, and future expectations. By using a focus group design, the perspectives of team members on the implementation process were discussed, which provided insights on a team level. However, the focus group design can also be regarded as a limitation, since some participants might have been reluctant to share their thoughts within a group. During some focus groups, the team leader, manager, and/or psychiatrist were present, and this hierarchy might have led to socially desirable answers. Also, despite the effort of the researcher (LZ) to foster an equal contribution of all participants, sometimes one or a few participants were dominant compared to others. A second limitation of this study concerns the selection of teams and care workers for the focus groups, as only one team per organization participated. Potentially, this may have led to a selection bias as the inclusion of the most enthusiastic team members may have resulted in more positive opinions and experiences regarding the implementation process in general and working with the ART model in particular. Furthermore, only the perspective of care workers, including peer workers and family peer workers, was included in this study. Future research should focus also on the perspectives of service users and significant others concerning the implementation of the ART model. Lastly, a more explicit comparison of the ART model and the WHO QualityRights initiative may be warranted to evaluate how both models may complement one another.

## 5. Conclusions

This study focused on factors influencing the implementation of the ART model. Three phases of implementation were identified. In the first phase, getting started, bottom-up and top-down support, information to all parties involved, and momentum were regarded as important. In the second phase, during the implementation process, team spirit, leadership and ambassadors, prioritizing team goals, sufficient tools and training, and dealing with structural limitations of large organizations were considered to require attention. For the third phase, striving for sustainability, dealing with setbacks, maintaining attention to the ART model, and exchange with other teams were seen as crucial. The findings of this study may contribute to the shift from traditional care approaches towards recovery-oriented care.

## Figures and Tables

**Table 1:** Principles of the ART model. Basis of Care and Support: Strengths, Needs, and Wishes of the Service User.

Active	Recovery	Triad
Active attitude	Health	Level of service user
	Identity	
Limited timeframe	Daily life	Level of team
Attention to personal wishes regarding treatment, support, and living	Community participation	Level of organization

**Table 2:** Participants per focus group.

Focus Group	Participants
1	1 nurse practitioner, 1 psychologist, 1 manager, 1 psychiatrist, 2 nurses
2	1 team leader, 1 peer worker, 1 family peer worker, 2 nurses, 2 social workers
3	1 manager, 1 psychiatrist, 3 nurses, 1 nurse practitioner
4	3 nurses, 2 social workers, 2 managers, 1 psychologist
5	1 manager, 1 care coordinator, 1 nurse practitioner, 1 peer worker, 2 nurses
6	1 team leader, 1 psychiatrist, 1 nurse, 2 social workers
7	2 social workers, 2 peer workers, 1 nurse practitioner, 1 manager
8	2 social workers, 1 psychologist, 3 nurses
9	1 nurse practitioner, 1 nurse, 1 psychologist, 2 social workers
10	1 team leader, 1 manager, 3 nurses, 1 peer worker, 1 psychologist
11	1 psychologist, 2 social workers, 1 policy advisor, 1 director
12	1 policy advisor, 1 team leader, 3 social workers
13	1 nurse practitioner, 2 nurses, 1 family peer worker, 1 manager, 1 peer worker
14	1 nurse, 1 psychiatrist, 1 social worker, 1 team leader, 1 ART project leader

**Table 3:** Description of the implementation process of team X.

Phase 1: Getting started One person within the organization of team X was closely involved in the development of the ART model. This person invited several professionals from the organization to contribute to the development of the model, among which were some members of team X. During the development of the ART model, they had already tried out the key principles into their own practice. For example, they discussed with service users and significant others that the stay would be limited. The implementation process was started with a large kick-off meeting, initiated by the management and board of directors. All teams in the organization that offered long-term care and support attended this meeting.
Phase 2: During implementation The management initiated several ways to exchange knowledge and experiences between the different teams in the organization. First, an ART working group with one or two members of every team discussed the implementation process of ART. Two members of team X actively took part in this working group. In addition, every six months, a large inspiration meeting was organized with all teams from the organization that implemented the ART model, to discuss aspects of the ART model, team goals regarding the implementation process of ART, and the progress of every team. In this way, the professionals were updated and inspired by the implementation process of other teams within the organization. Furthermore, the majority of the team members attended national conferences on the ART model.
Some months after the start of the implementation, team X faced some major changes. The management initiated a reallocation of the service users over the different wards of the location, based on the stage of their recovery process. The number of beds on institutional grounds needed to be decreased and the ward of team X needed to change from a closed setting to an open setting. Every week, the team had a meeting to discuss the practical arrangements for this change in order to make it a success. According to the team members, the change from a closed to an open setting provided a boost to the implementation of the ART model, because they needed to focus on the recovery process of the service users even more.
Phase 3: Striving for sustainability Later in the process, the organization struggled with financial problems. In addition, not every worker in the team supported the ART model, so some of the team members eventually left team X for this reason. As a result of the financial situation, there were fewer possibilities to attract new employees. Due to the shortage of personnel, the feeling of safety was at stake for the professionals in the team, and they did not feel they had space to focus on the implementation of the ART model. For example, extra activities (e.g., extra training) were not possible at this time. Therefore, professionals of team X and two other teams of the organization that struggled with the same issue suggested reallocating the team members and service users from three wards to two wards. At this time, a new manager started, who was willing to take some risk and actively started to recruit new employees, despite the financial situation of the organization. It turned out to be difficult to find professionals who were willing to work in the long-term setting. Yet, this manager was successful in attracting new team members by demonstrating the creativity one needs to have to work on recovery with service users in this setting. Currently, team X is stable and professionals experience the freedom and creativity to implement the ART model. Every six months, the team members evaluate the goals they have set and decide upon new goals regarding implementation. The management of the organization plans to hold internal audits, to keep track of the implementation process.

## Data Availability

The data of this study are saved at the Amsterdam UMC and are accessible to members of the research team. For questions regarding the data retrieved in this study, the reader can contact the first author (L.Z.).

## Supplementary Materials

The following supporting information can be downloaded at: https://www.mdpi.com/article/10.3390/jcm13123488/s1, Supplementary file S1: Topic list, Supplementary file S2: Coding tree.
