# Supplementary material for: From Model to Practice: A Qualitative Study on Factors Influencing the Implementation of the Active Recovery Triad (ART) Model in Long-Term Mental Health Care
Source: J Clin Med. 2024 Jun 14;13(12):3488. doi: 10.3390/jcm13123488 (PMC11205107; doi:10.3390/jcm13123488)
Supplement: Supplementary file 1 [file jcm-13-03488-s001.zip › jcm-2896683-supplementary.pdf]

Supplementary material

S1: Topic list

- When did you start the implementation process? How long have you been working on it?
- How did the ART model find its way to the team/ward?
- How did you approach the start of the implementation?
- Where are you now?
- What is your view on the progress of the implementation process within your team?
- What are your plans on proceeding the implementation process?
- What are you proud of? Do you have an example of a best practice?
- Which elements contribute to a better implementation?
- What hampering factors did you encounter in the process? How did you overcome this?
- Do you notice resistance during the implementation? If yes, where does that resistance come from? How can you overcome this resistance?
- What do you hope to achieve with the implementation of ART?
- How does working with ART affect how service users are approached/supported?
- What is the most striking change you see since working with ART?
- Do you already notice the first effects of ART? For example in the recovery process of service users?
- What would you advise to other teams that want to start implementing the ART model?

S2: Coding tree of thematic analysis

Phase 1: Getting started

- Theme 1: Support from the management and the work floor
    - Support from management
    - Introduction of ART within the organization
    - Initiative from the workfloor
    - Care workers involved in the development process of the ART model
- Theme 2: Information to care workers, service users and family
    - Unclarity
    - ART handbook
    - Presentation about ART
    - Involvment of family
    - Explanation to service users
    - Kick off meetings
    - ART is not new
    - Insecurity and resistance
- Theme 3: Creating momentum
    - Clear starting point
        - Kick off meetings

        - No clear starting point
    - Team building days
    - First ART audit
    - Momentum created by management

Phase 2: During implementation

- Theme 4: A stable team with a good spirit
    - Personnel
        - Shortage of staff / enough care workers in the team
        - Attract new personnel
        - Older employees / new young staff
    - Team atmosphere
        - Communication
        - Connection between team members
        - Good cooperation
        - Positivity
        - Reflection
        - Invest in team spirit
        - Everyone on board
- Theme 5: Leadership and ambassadors
    - Mutual trust
    - Feeling of encouragement
    - Time for implementation
    - On the same page
    - ART project group
    - Ambassadors
        - Auditors
        - Project leader
        - Selected ambassadors
        - Cooperation between ambassadors and rest of team
- Theme 6: Setting and prioritizing goals
    - Goal setting by care workers in the team
    - Evaluating team goals
    - Clear/unclear implementation process
        - Structure
        - Transparency
        - Implementation plan
    - Small goals
    - Not everything at once
        - ART monitor – select three items to start with
- Theme 7: Tools and training
    - Training on recovery-oriented care

        - Training on recovery interventions
        - Training on ART
        - Gap between training and daily work
    - ART monitor, ART handbook
    - Coaching on the job
    - Tools and interventions available
- Theme 8: Changing structures of large organizations
    - Takes time to adapt the whole organization
    - Old housing facilities
    - Environment as bottleneck
    - ICT facilities
        - Clinical records

Phase 3: Striving for sustainability

- Theme 9: Dealing with setbacks
    - Open vacancies for a long time
    - Shortage of accommodations to move out to
    - Financial situation of organization
    - Administration workload
    - Creativity
    - What gives energy?
        - Celebrating small successes
        - Service users motivating each other
- Theme 10: Maintaining attention to the ART model
    - Lose track of implementation process
    - Audit
    - Structural meetings about ART
    - Contact with other teams
    - National meetings
- Theme 11: Not alone but together
    - National developments
    - Contact with other organizations
    - National meetings – platform meetings and conferences
    - Contact with other teams in the organization
    - Part of a leaning network
